# Quadratic function between arterial partial oxygen pressure and mortality risk in sepsis patients: an interaction with simplified acute physiology score

**DOI:** 10.1038/srep35133

**Published:** 2016-10-13

**Authors:** Zhongheng Zhang, Xuqing Ji

**Affiliations:** 1 Department of emergency medicine, Sir Run-Run Shaw Hospital, Zhejiang University School of Medicine, Hangzhou, 310016, China; 2 Department of critical care medicine, Jinhua municipal central hospital, Jinhua hospital of Zhejiang university, Zhejiang, P.R.China

## Abstract

Oxygen therapy is widely used in emergency and critical care settings, while there is little evidence on its real therapeutic effect. The study aimed to explore the impact of arterial oxygen partial pressure (PaO_2_) on clinical outcomes in patients with sepsis. A large clinical database was employed for the study. Subjects meeting the diagnostic criteria of sepsis were eligible for the study. All measurements of PaO_2_ were extracted. The primary endpoint was death from any causes during hospital stay. Survey data analysis was performed by using individual ICU admission as the primary sampling unit. Quadratic function was assumed for PaO_2_ and its interaction with other covariates were explored. A total of 199,125 PaO_2_ samples were identified for 11,002 ICU admissions. Each ICU stay comprised 18 PaO_2_ samples in average. The fitted multivariable model supported our hypothesis that the effect of PaO_2_ on mortality risk was in quadratic form. There was significant interaction between PaO_2_ and SAPS-I (p = 0.007). Furthermore, the main effect of PaO_2_ on SOFA score was nonlinear. The study shows that the effect of PaO_2_ on mortality risk is in quadratic function form, and there is significant interaction between PaO_2_ and severity of illness.

Oxygen therapy is widely used in emergency and critical care settings, aiming to prevent and/or correct hypoxemia and hypoxia^1^. Tissue hypoxia is a common and devastating condition occurring in various medical conditions including infection, trauma, acute respiratory distress syndrome and circulatory shock. If not promptly treated, tissue hypoxia may further lead to multiple organ dysfunction syndrome that has been associated with significantly increased risk of death. As a result, most critical care specialists prefer early initiation of oxygen therapy, even without documented hypoxia.Furthermore, clinical guidelines seldom recommend the titration of oxygen therapy to prevent potential hyperoxemia^2^,^3^. Such situation is a result of the underestimation of adverse effect of hyperoxemia, and the side effect may not be directly observable in clinical setting.

There is a few evidence showing the association between hyperoxia and tissue damage. Short-term hyperoxia (<10 min) during anesthesia induction has been associated with the development of atelectasis^4^,^5^. For prolonged hyperoxia, inflammatory changes in the airway membrane are noted^6^. However, there is no empirical data showing the association of hyperoxia and mortality in the intensive care unit (ICU). Patients with sepsis are at increased risk of hypoxia because of the well-known gap between oxygen supply and oxygen demand^7^. On the other hand, septic patients are prone to the adverse effect of hyperoxia. Some animal studies have shown that oxygen therapy greatly influences the progression and clinical manifestation of sepsis-induced multiple system organ dysfunction^8^, while others reported conflicting results^9^,^10^. The underlying mechanisms for such disparity remain largely unknown based on current evidence but we proposed that different degrees of illness severity might have impact on the effect of oxygen therapy. The present study aimed to investigate the association between hyperoxia and ICU mortality in patients with sepsis. We hypothesized that (1) the effect of arterial oxygen pressure (PaO_2_) on mortality followed a quadratic form that both low and high PaO_2_ were associated with high mortality risk; (2) there is an interaction between PaO_2_ and severity of illness.

## Methods

### Database and data extraction

We employed the critical care big data named Multiparameter Intelligent Monitoring in Intensive Care II (MIMIC-II) for the study. The latest version of MIMIC-II was version 2.6, which comprised more than 30000 ICU admissions. The database contained all clinical information during ICU stay, including demographics, laboratory findings, imaging studies, vital signs and progress notes were available^11^,^12^. The establishment of MIMIC- II was approved by the Institutional Review Boards of the Massachusetts Institute of Technology (Cambridge, MA) and Beth Israel Deaconess Medical Center (Boston, MA). De-identification was performed to ensure patients’ confidentiality. Our access to the database was approved after completion of the NIH web-based training course named “Protecting Human Research Participants” by the author Z.Z. (certification number: 1132877). Informed consent was waived because this was a study of clinical database.

Clinical information on demographics (age, gender), care unit types, physiological variables (e.g. hear rate, temperature, mean arterial blood pressure), white blood cells, ICD-9 diagnosis, microbiological results and PaO_2_ were extracted. The severity of illness was represented by simplified acute physiology score (SAPS-I) and sequential organ failure assessment (SOFA) scores. In the database, blood oxygen pressure was recorded without denoting whether it was venous or arterial. In patients with sepsis and/or septic shock it is common to measure both central venous and arterial blood gas. In order to distinguish between venous and arterial blood gas, we screened consecutive blood gas samples measured within 10 minutes. The difference of oxygen pressure between the two measurements should more than 20 mmHg. The one with oxygen pressure <80 mmHg was considered to be the venous oxygen pressure. As a result, a total of 4222 samples were considered to be venous oxygen pressure. The mean value of venous oxygen pressure was 36 mmHg (95% CI: 28–56 mmHg).

### Inclusion criteria

Subjects meeting the diagnostic criteria of sepsis were identified from the database. Sepsis was defined according to that defined in Surviving sepsis Campaign^13^. SIRS was defined as more than one of the following criteria within 24 hours after ICU admission: 1) fever (>38.3 °C) or hypothermia (<36 °C); 2) tachycardia (>90/min); (3) leukocytosis (WBC count > 12000/μL) or leukopenia (WBC count < 4000/μL); (4) tachypnea (>20/min). If there were multiple records during that time period, the one most likely to fulfill the criteria was adopted (e.g. highest or lowest temperature, highest heart rate). Infection was defined as documented or suspected. Infection was defined in the database as fulfilling one of the following criteria (1) ICD9 contains the term “infection” or “pneumonia”; (2) microbiological culture was positive. The Stata codes for extracting sepsis patients from the database were described elsewhere^14^.

### Study endpoint

The primary endpoint was death from any causes during hospital stay. Secondary endpoint was the maximum SOFA and the length of stay in ICU.

### Statistical analysis

Categorical variables were expressed as the number and percentage, and their differences between survivors and non-survivors were compared using Chi-square test. Continuous variables were expressed as mean and standard deviations (SD) and compared by using t test^15^.

Because measurements of arterial blood gas was repeated several times for each subjects, survey data analysis was performed using individual ICU admission as the primary sampling unit. There was no stratification.

The purpose of the present study was to explore the association of PaO_2_ with mortality outcome, thus we forced all variables into the model for risk adjustment. These selected variables (e.g. age, sex, serum lactate, SOFA score, SAPSI, care unit type) are well documented as a risk factor for mortality^16^,^17^. To make full use of data information, missing values were replaced with the mean value of that variable^18^. For example, missing serum lactate was replaced by the mean value of 2.76 mmol/l. Because we hypothesized that PaO_2_ followed a U-shaped relationship with mortality risk, a quadratic form function was assumed for model specification. Our interests are to estimate the coefficients for the quadratic and linear term of PaO_2_. A quadratic function allows for one turning point at which the value of PaO_2_ corresponds to lowest morality rate. After determination of the quadratic main effect of PaO_2_, interactions between PaO_2_ and other variables were investigated. We employed a conservative p = 0.01 for inclusion of interaction terms. Graphical examination of the interaction effect was performed to give subject audiences a visual impression of how the effect of PaO_2_ changed with different levels of severity of illness. Goodness-of-fit test of the fitted model was examined by using F-adjusted mean residual test after survey data analysis^19^. Because the severity of illness scores could be treated as continuous variables, a multivariable linear regression model was built to investigate the association of PaO_2_ and the maximum SOFA score.

All statistical analyses were performed by using Stata 13.1 (StataCorp, College Station, Texas 77845 USA). A level of p < 0.05 was considered statistically significant.

## Results

**Figure 1:**
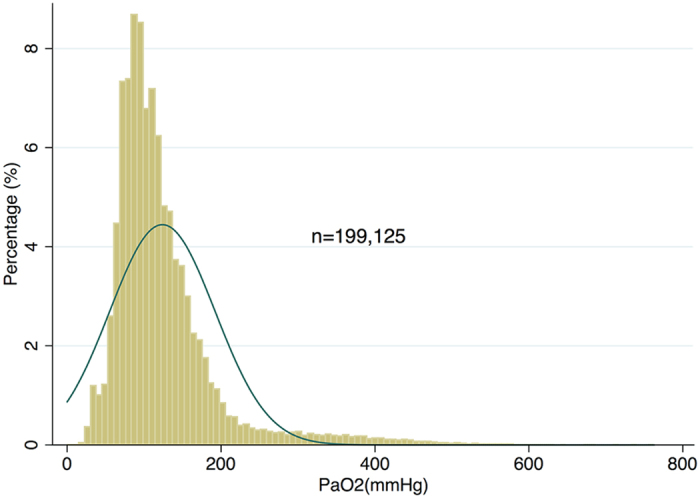
Histogram showing the distribution of arterial oxygen partial pressure (PaO_2_) in our cohort (n = 199,125). It appears that PaO_2_ is approximately normally distributed with the mean value around 100 mmHg.

There are 28432 ICU admissions met the criteria of SIRS, among which only 11002 had both positive culture and partial pressure of arterial oxygenation measured. A total of 199,125 PaO_2_ samples were identified for 11,002 ICU admissions. Each ICU stay comprised 18 PaO_2_ samples in average. The distribution of PaO_2_ was shown in Fig. 1, which is nearly normal with an average around 120 mmHg (95% CI: 44–317 mmHg). There were 9352 survivors and 1650 non-survivors with a mortality rate of 15% (Table 1). Survivors showed significantly higher values of PaO_2_ than non-survivors (175.41 ± 125.42 vs. 157.30 ± 118.27 mmHg, p < 0.001). As expected, non-survivors were significantly older than survivors (69.3 ± 16.1 vs. 63.9 ± 20.0 years, p < 0.001). Serum lactate was also higher in non-survivors than in survivors (3.69 ± 3.31 vs. 2.45 ± 2.020020 mmol/l, p < 0.001). SAPS-I (19.32 ± 5.54 vs. 15.50 ± 4.92, p < 0.001) and SOFA (10.11 ± 4.51 vs. 7.04 ± 3.73, p < 0.001) were both significantly higher in non-survivors than in survivors. Overall, the care unit type was significantly different between survivors and non-survivors. Patients from MICU were more likely to die than those from other care unit types (51% vs. 41%, p < 0.05). There was no significant difference in sex between survivors and non-survivors.

**Figure 2:**
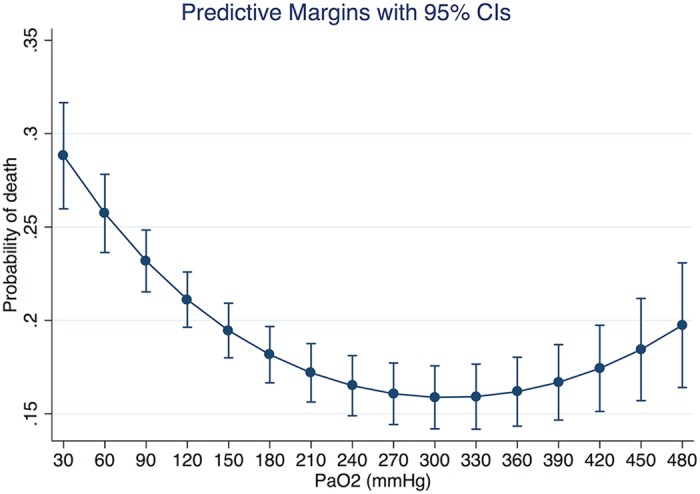
Marginal effect of PaO_2_ on mortality risk after adjustment for other covariates (all variables finally remained in the model) . The probability of death decreases with increasing PaO_2_, and reaches a nadir at a PaO_2_ value of 300 mmHg. Thereafter, the probability of death rises with increasing PaO_2_.

**Figure 3:**
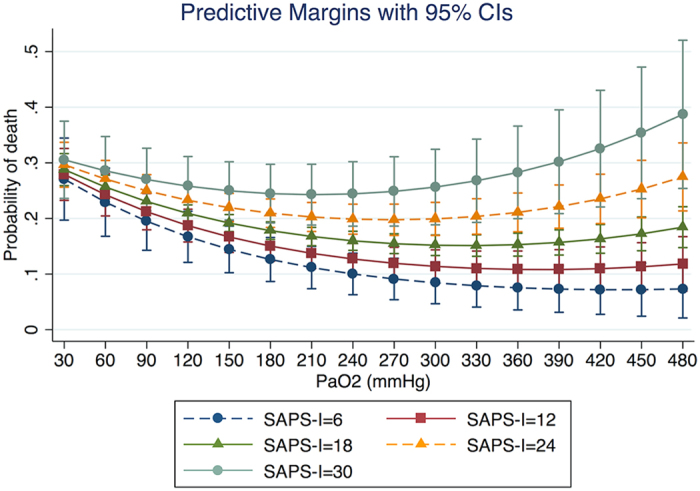
The relationship between PaO_2_ and mortality risk at different levels of severity of illness. Covariates except for SOFA were adjusted for in each curve. As expectedly, patients with SAPS-1 = 30 showed highest risk of death and the nadir of the curve was at the PaO_2_ value of 210 mmHg. In contrast, those with SAPS-1 = 6 showed the lowest risk of death across the entire PaO_2_ range, and the curve reaches a nadir at the PaO_2_ value of 420 mmHg.

The fitted model supported our hypothesis that the effect of PaO_2_ on mortality risk was in quadratic form (Table 2). The odds ratio for the quadratic term was 1 (p < 0.001). Also there was significant interaction between PaO_2_ and SAPS-I (p = 0.007). Other potential interaction terms were not statistically significant and were excluded from the model. Covariates entered into the model were quadratic term of PaO_2_ (OR: 1.000, 95% CI: 1.000–1.000), linear term of PaO_2_ (OR: 0.990, 95% CI: 0.987–0.993), age (OR: 1.015, 95% CI: 1.009–1.020), lactate (OR: 1.081, 95% CI: 1.039–1.125), CSRU (OR: 0.474, 95% CI: 0.376–0.598), and SOFA (OR: 1.075, 95% CI: 1.042–1.109). The area under curve of the model was 0.84. Figure 2 shows the marginal effect of PaO_2_ on mortality risk after adjustment for other covariates. In the range of less than 300 mmHg, increasing PaO_2_ was associated with reduced risk of death. However, in the range of more than 300 mmHg increasing PaO_2_ was associated with increased risk of death. The nadir of mortality risk was at a PaO_2_ value of approximately 300 mmHg. Figure 3 shows the relationship between PaO_2_ and mortality stratified by severity of illness. At SASP-I = 6, the nadir of mortality risk occurred at a PaO2 value of 450 mmHg, after which the mortality increased. However, at SASP-I = 30 the nadir of mortality risk occurred at a PaO_2_ value of 200 mmHg.

**Figure 4:**
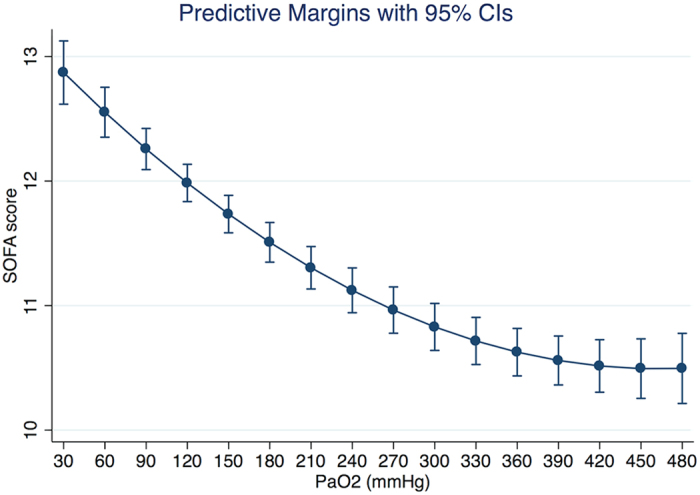
Marginal effect of PaO_2_ on SOFA score after adjustment for other covariates (all variables finally remained in the model). The SOFA score decreased monotonously with increasing PaO_2_ before 450 mmHg. After that further increase in PaO_2_ has no effect on reducing SOFA.

The maximum Organ failure score was the secondary endpoint reported in our analysis. Multivariable linear regression model was fitted. Again the main effect of PaO_2_ on SOFA score was nonlinear. The coefficient of the quadratic term was statistically significant (Table 3). The SOFA score increased monotonously with increasing PaO_2_ before 450 mmHg. After that further increase in PaO_2_ has no effect on reducing SOFA (Fig. 4).

## Discussion

The study showed a significant quadratic term for the effect of PaO_2_ on mortality risk, indicating that hyperoxia is harmful at high levels. Furthermore, there was interaction between PaO_2_ and simplified physiological score, with greater negative effect of hyperoxia in patients with higher SASP-I scores. The study focused on patients with sepsis and organ failure score was employed as the secondary outcome. In our study the maximum SOFA score showed a negative correlation with PaO_2_, but at PaO_2_ > 450 mmHg the relationship disappeared. With large clinical database and sophisticated model building technique, our data support the notion that hyperoxia is harmful in critically ill patients with sepsis.

In a recent study involving patients with cardiac arrest, Elmer J. and coworkers^20^ reported that severe hyperoxia (defined as PaO_2_ > 300 mmHg) was associated with increased risk of death in both unadjusted and adjusted approaches. It is surprising that the cutoff points for negative effect of hyperoxia are so consistent in both studies, although they included different kind of patient populations. However, Elmer’s study failed to identify the association between moderate or probable hyperoxia (PaO_2_: 101–299 mmHg) and improved survival. This can be partly explained by the small sample size (n = 184) in their study, which lacks statistical power to detect small effect size. In our study, moderate hyperoxia was associated with significantly improved survival, and the relationship between PaO_2_ and risk of death was monotonically decreasing at PaO_2_ < 300 mmHg. Other studies involving cardiac arrest patients yielded conflicting results^21^,^22^,^23^. Rodríguez-González R and coworkers have investigated the effect of hyperoxia on organ dysfunction in experimental model of sepsis^8^. The result showed that hyperoxia had negative impact on sepsis-induced organ dysfunction. They further proved that the negative impact was mediated via inflammatory cytokines and reactive oxygen species. However, the result was not replicated in human studies. Our study showed that SOFA decreased with increasing PaO_2_, but the decreasing rate (slope) was reduced at higher levels of PaO_2_. There is a few evidence suggesting beneficial effects of hyperoxia on inflammatory response, providing support to our findings^9^. With respect to the mortality outcome, Stolmeijer R and coworkers reported that hyperoxia was associated with increased risk of death in sepsis patients, which is consistent with our findings^24^. However, they reported inspired oxygen saturation but not PaO_2_, which hampered further comparison with our study.

One important indication of our study is that higher levels of oxygen can be given to patients with sepsis. In convention, it is recommended to use the least inspiratory O_2_ fraction associated with an arterial O_2_ tension of 55 to 80 mm Hg or an arterial oxygen saturation of 88 to 95% 25. Our data showed that the risk of death decreases monotonously at the PaO_2_ values below 300 mmHg, suggesting additional benefits by increasing PaO_2_ to 300 mmHg. Further increment in PaO_2_ beyond 300 mmHg will exert negative impact on survival. Probably, the negative pathophysiological effect of hyperoxia found in experimental studies outweighs its beneficial effect at such high PaO_2_ levels^26^. On the other hand, because causal inference cannot be made in our study, it is also probable that it is the severity of illness that result in inability of the lung to transport more oxygen to arterial blood. For instance, severe acute respiratory distress syndrome with hypoxemia is a prognosticator of adverse outcome. PaO_2_ cannot be elevated even with aggressive treatment. Another implication of the study is that sepsis patients with higher SASP-I score are more subject to the negative impact of hyperoxia. This is somewhat contradictory to conventional ideas that more oxygen should be given to more critically ill patients. Because the interaction effect in our study used relatively conservative p value, it should be reliable and robust to further test.

The strength of the study was the use of a large clinical database. A large sample size allows for complex model building strategy with many degrees of freedom. To make our result robust, especially for interaction effect, we used conservative p value of 0.01 as the significance level. The result showed that there was interaction between PaO_2_ and severity of illness. Several limitations need to be acknowledged in the study. First, the selection of sepsis subjects based on data mining was not straightforward as can be done in prospective design. The diagnosis of sepsis was not incorporated into ICD-9 and we had to extract vital signs and information on possible infection to confirm the existence of sepsis. The diagnosis of SIRS can be accurate based on documented physiological signs, but infection was somewhat illusive. For example, the confirmation of infection relies partly on microbiological findings. Sometimes the positive culture result may be colonization instead of true infection. However, this method has been fully discussed by experts, reflecting the best way to define sepsis based on data extracted from electronic medical record system. Second, the study was retrospective in design and was subject to selection bias. For instance, the physician may not order arterial blood gas for patients with better conditions. Exclusion of this group of patients results in potential selection bias. Third, it is well known that sepsis combined with shock is associated with increased risk of death, and this subgroup of patients is subject to imbalance between oxygen demand and supply. It is technically challenging to distinguish between patients with and without shock. However, we used surrogate lactate to represent tissue hypoxia, which after inclusion into the model showed significant association with mortality. Forth, oxygen therapy was not fully explored in the study. There are varieties of techniques to improve oxygenation in ICU, including high-flow nasal oxygen therapy, mechanical ventilation and even extracorporeal membrane oxygenation. However, including these interventions into analysis is technically challenging. At the start point, we hypothesized that no matter what kind of intervention is employed, the ultimate goal is to improve PaO_2_.

In conclusion, the study shows that the effect of PaO_2_ on mortality risk is in quadratic function form. The increment in PaO_2_ was associated with reduced risk of death with PaO_2_ < 300 mmHg. Thereafter, the increase in PaO_2_ beyond 300 mmHg was associated with increased risk of death. Due to retrospective nature of the study, further experimental trials are needed to confirm our result.

## Additional Information

**How to cite this article**: Zhang, Z. and Ji, X. Quadratic function between arterial partial oxygen pressure and mortality risk in sepsis patients: an interaction with simplified acute physiology score. *Sci. Rep*. **6**, 35133; doi: 10.1038/srep35133 (2016).

## Figures and Tables

**Table 1:** Characteristics of included patients.

	Total (n = 11002)		Survivors (n = 9352)		Non-survivors (n = 1650)		P value
	Mean	SD	Mean	SD	Mean	SD	
PaO_2_	172.69	124.54	175.41	125.42	157.30	118.27	<0.001
Age	64.74	19.53	63.94	19.96	69.32	16.14	<0.001
Lactate	2.67	2.34	2.45	2.02	3.69	3.31	<0.001
SAPS-I	16.06	5.19	15.50	4.92	19.32	5.54	<0.001
SOFA	7.50	4.01	7.04	3.73	10.11	4.51	<0.001
Care unit	Number	Fraction	Number	Fraction	Number	Fraction	<0.001
MICU	4715.00	0.43	3876.00	0.41	839.00	0.51	.
SICU	686.00	0.06	613.00	0.07	73.00	0.04	.
CCU	2208.00	0.20	1819.00	0.19	389.00	0.24	.
CSRU	3393.00	0.31	3044.00	0.33	349.00	0.21	.
Sex (male, %)	6052.00	0.55	5149.00	0.55	903.00	0.55	0.80

Abbreviations: MICU: medial intensive care unit; SICU: surgical intensive care unit; CCU: coronary care unit; CSRU: Cardiac Surgery Recovery Unit; SAPS-I: simplified acute physiology score; SOFA: sequential organ failure assessment.

**Table 2:** Multivariable logistic regression model with interaction term.

	Odds ratio	Standard error	Lower limit of 95% CI	Upper limit of 95% CI	p
Age	1.015	0.003	1.009	1.020	<0.001
Lactate	1.081	0.022	1.039	1.125	<0.001
Sex (male as reference)	0.863	0.084	0.713	1.043	0.128
Care unit (MICU as reference)	1.000	.	1.000	1.000	.
SICU	0.819	0.174	0.540	1.243	0.349
CCU	0.959	0.118	0.753	1.221	0.733
CSRU	0.474	0.056	0.376	0.598	<0.001
PaO_2_	0.990	0.002	0.987	0.993	<0.001
PaO_2_ × 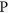 aO_2_	1.000	0.000	1.000	1.000	<0.001
SAPS-I	1.002	0.016	0.972	1.033	0.901
PaO_2_ × SAPS-I	1.000	0.000	1.000	1.000	0.007
SOFA	1.075	0.017	1.042	1.109	<0.001
Constant	0.102	0.033	0.054	0.193	<0.001

Abbreviations: MICU: medial intensive care unit; SICU: surgical intensive care unit; CCU: coronary care unit; CSRU: Cardiac Surgery Recovery Unit; SAPS-I: simplified acute physiology score; SOFA: sequential organ failure assessment. Goodness-of –fit test: F(9,10287) = 1.48; Prob > F = 0.1499 There was no interaction between PaO_2_ × age, and between PaO_2_ × SOFA. PaO_2_ was incorporated as quadratic term.

**Table 3:** Multivariable linear regression model using the maximum SOFA score as the outcome variable.

	Coefficient	Standard error	Lower limit of 95% CI	Upper limit of 95% CI	p
Age	−0.018	0.005	−0.027	−0.009	<0.001
Lactate	0.345	0.033	0.280	0.410	<0.001
Sex (female)	−0.879	0.149	−1.171	−0.588	<0.001
MICU	0				
SICU	−1.803	0.306	−2.402	−1.203	<0.001
CCU	0.466	0.215	0.044	0.888	0.031
CSRU	0.062	0.174	−0.280	0.403	0.724
PaO_2_	−0.012	0.001	−0.015	−0.009	<0.001
PaO_2_ × PaO_2_	0.000	0.000	0.000	0.000	<0.001
SAPS−I	0.260	0.017	0.227	0.293	<0.001
Constant	9.045	0.443	8.176	9.914	<0.001

Abbreviations: MICU: medial intensive care unit; SICU: surgical intensive care unit; CCU: coronary care unit; CSRU: Cardiac Surgery Recovery Unit; SAPS-I: simplified acute physiology score; SOFA: sequential organ failure assessment.
